# II BRAZILIAN CONSENSUS ON GASTRIC CANCER BY THE BRAZILIAN GASTRIC CANCER ASSOCIATION

**DOI:** 10.1590/0102-672020190001e1514

**Published:** 2020-08-24

**Authors:** Leandro Cardoso BARCHI, Marcus Fernando Kodama Pertille RAMOS, André Roncon DIAS, Nelson Adami ANDREOLLO, Antônio Carlos WESTON, Laércio Gomes LOURENÇO, Carlos Alberto MALHEIROS, Paulo KASSAB, Bruno ZILBERSTEIN, Consensus, Álvaro Antônio Bandeira Ferraz, Amir Zeide Charruf, André Brandalise, André Maciel da Silva, Barlon Alves, Carlos Augusto Martinez Marins, Celso Vieira Leite, Claudio José Caldas Bresciani, Daniel Szor, Donato Roberto Mucerino, Durval R. Wohnrath, Elias Jirjoss Ilias, Euclides Dias Martins, Fabio Pinatel Lopasso, Felipe José Fernandez Coimbra, Fernando E. Cruz Felippe, Flávio Daniel Saavedra Tomasisch, Flavio Roberto Takeda, Geraldo Ishak, Gustavo Andreazza Laporte, Herbeth José Toledo Silva, Ivan Cecconello, Joaquim José Gama Rodrigues, José Carlos Del Grande, Leonardo Milhomem da Motta, Leonardo Rocha Ferraz, Luis Fernando Moreira, Luis Roberto Lopes, Marcelo Garcia Toneto, Marcelo Mester, Marco Antônio Gonçalves Rodrigues, Marineide Prudêncio de Carvalho, Maurice Youssef Franciss, Nora Manoukian Forones, Oly Campos Corletta, Osmar Kenji Yagi, Osvaldo Antonio Prado Castro, Osvaldo Malafaia, Paulo Pimentel Assumpção, Paulo Roberto Savassi-Rocha, Ramiro Colleoni, Rodrigo Jose de Oliveira, Rubens Antonio Aissar Sallun, Rui Weschenfelder, Saint Clair Vieira de Oliveira, Thiago Boechat de Abreu, Tiago Biachi de Castria, Ulysses Ribeiro, Williams Barra, Wilson Luiz da Costa, Wilson Rodrigues de Freitas

**Affiliations:** 1 Hospital das Clínicas, Faculty of Medicine, University of São Paulo, São Paulo, SP, Brazil; 2 Faculty of Medicine São Leopoldo Mandic, Campinas, SP, Brazil; 3 State University of Campinas, Campinas, SP, Brazil; 4 Department of Surgery, Santa Casa de Porto Alegre, Porto Alegre, RS, Brazil; 5 Federal University of São Paulo, São Paulo, SP, Brazil; 6 Department of Surgery, Santa Casa de São Paulo, São Paulo, SP, Brazil

**Keywords:** Gastric neoplasms, Gastric cancer, Gastrectomy, Lymphadenectomy, Consensus, Adenocarcinoma, Neoplasias gástricas, Câncer gástrico, Gastrectomia, Linfadenectomia, Consenso, Adenocarcinoma

## Abstract

**Background:**

Since the publication of the first Brazilian Consensus on Gastric Cancer (GC) in 2012 carried out by the Brazilian Gastric Cancer Association, new concepts on diagnosis, staging, treatment and follow-up have been incorporated.

**Aim:**

This new consensus is to promote an update to professionals working in the fight against GC and to provide guidelines for the management of patients with this condition.

**Methods:**

Fifty-nine experts answered 67 statements regarding the diagnosis, staging, treatment and prognosis of GC with five possible alternatives: 1) fully agree; 2) partially agree; 3) undecided; 4) disagree and 5) strongly disagree A consensus was adopted when at least 80% of the sum of the answers “fully agree” and “partially agree” was reached. This article presents only the responses of the participating experts. Comments on each statement, as well as a literature review, will be presented in future publications.

**Results:**

Of the 67 statements, there was consensus in 50 (74%). In 10 declarations, there was 100% agreement.

**Conclusion:**

The gastric cancer treatment has evolved considerably in recent years. This consensus gathers consolidated principles in the last decades, new knowledge acquired recently, as well as promising perspectives on the management of this disease.

## RESUMO

**Racional:**

Desde a publicação do primeiro Consenso Brasileiro sobre Câncer Gástrico em 2012 realizado pela Associação Brasileira de Câncer Gástrico (ABCG), novos conceitos sobre o diagnóstico, estadiamento, tratamento e seguimento foram incorporados.

**Objetivo:**

Promover uma atualização aos profissionais que atuam no combate ao câncer gástrico (CG) e fornecer diretrizes quanto ao manejo dos pacientes portadores desta afecção.

**Métodos:**

Cinquenta e nove especialistas responderam 67 declarações sobre o diagnóstico, estadiamento, tratamento e prognóstico do CG com cinco alternativas possíveis: 1) concordo plenamente; 2) concordo parcialmente; 3) indeciso; 4) discordo e 5) discordo fortemente. Foi considerado consenso a concordância de pelo menos 80% da soma das respostas “concordo plenamente” e “concordo parcialmente”. Este artigo apresenta apenas as respostas dos especialistas participantes. Os comentários sobre cada declaração, assim como uma revisão da literatura serão apresentados em publicações futuras.

**Resultados:**

Das 67 declarações, houve consenso em 50 (74%). Em 10 declarações, houve concordância de 100%.

**Conclusão:**

O tratamento do câncer gástrico evoluiu consideravelmente nos últimos anos. Este consenso reúne princípios consolidados nas últimas décadas, novos conhecimentos adquiridos recentemente, assim como perspectivas promissoras sobre o manejo desta doença.

## INTRODUCTION

The incidence of gastric cancer (GC) has decreased around the world. Once the second most common type of cancer, currently CG is the fifth, after lung, breast, prostate and colorectal^6^ ^,^ ^10^ ^,^ ^15^. Its incidence has dropped in the last 50 years due to the improvement in basic sanitation conditions, the use of refrigerators and consumption improvement of fresh fruits and vegetables and less salt intake, which was then widely used to preserve food. Other factors that contributed to this decline were the eradication of *Helicobacter pylori* and the screening intensification in several countries^14^.

Despite that, the mortality rate remains high. In 2018, CG recovered the second place in cancer deaths around the world, surpassing liver cancer again and placing only after lung cancer^6^. In Brazil, it is the third most common type among men and the fifth among women^1^. According to the *Instituto Nacional do Câncer* (INCA) data, it is estimated that in the 2018/2019 biennium 21,290 new cases were diagnosed (13,540 in men and 7,750 in women) and about 15,000 deaths in 2017 related to it^11^. 

Adenocarcinoma is the most common histological type, accountable for about 90-95% of cases. Other less common malignant neoplasms of the stomach such as lymphoma (4%), neuroendocrine tumor (3%) and gastrointestinal stromal tumor (GIST) will not be addressed in this consensus^5^ ^,^ ^9^.

Due to the continental dimension of Brazil, the incidence, management and prognosis varies widely according to the different regions of the country. In 2013, the first Brazilian Consensus on Gastric Cancer was published by the Brazilian Gastric Cancer Association (BGCA). This consensus aimed to unify and standardize the diagnosis and management of this condition^21^. Undoubtedly, the publication of this guideline made it possible to spread knowledge among medical professionals and, consequently, improve care and increase survival of these patients. 

However, since the publication of the first Consensus, many aspects related to CG have changed^16^ ^,^ ^17^. It lists: new TNM staging classification was implemented by the American Joint Committee on Cancer and Union for International Cancer Control (AJCC/UICC)^18^; the agreement between the West and the East on the pivotal role of D2 lymphadenectomy as a standard surgical treatment; the increasing role of multimodal therapy (neoadjuvant, perioperative and adjuvant chemotherapy/radiotherapy); the replacement of the systematic removal of the lymph node stations by the minimum number of 15 lymph nodes in the D2 lymphadenectomy; the indication for multivisceral resection (splenectomy); the endoscopic treatment in early GC and the role of minimally invasive surgery (laparoscopic or robotic) as an alternative surgical approach. 

Thus, in view of the evidence gathered in recent years, it is opportune to update the Brazilian guidelines on CG through a new consensus. It is important to note that the guidelines hereby presented are not arbitrary and, therefore, it is up to each medical assistant/multidisciplinary team to adopt the best conduct according to local reality and the resource availability, as long as they are indeed beneficial to the patient. 

## METHODS

The Brazilian Gastric Cancer Association celebrated 20 years of its foundation in 1999. To solemnize this date, a commemorative day was held in Porto Alegre (RS) on August 16, when there was the opportunity to debate and present the results of this new Consensus. Three months before this event, a group of ABCG experts created 67 GC statements on diagnosis, staging, treatment and prognosis with five possible alternatives: 1) fully agree; 2) partially agree; 3) undecided; 4) disagree and 5) strongly disagree. These statements were sent to 59 specialists in GC treatment from all regions of Brazil (surgeons, oncologists, endoscopists, pathologists, etc.), embracing more than 20 universities institutions. The participants were able to mark only one answer. A consensus was achieved when the sum of “fully agree” and “partially agree” reached at least 80%. This article presents only the responses of the participating experts. Comments on each statement, as well as a literature review, will be presented in future publications.

## RESULTS

Of the 67 statements presented, a consensus was reached in 50 (74%). In 10 statements, there was 100% agreement.

### Diagnosis statements

####  Statement 1 

The main method of gastric cancer diagnosis is through an upper gastrointestinal endoscopy with biopsy. The endoscopic examination report must contain precise information regarding location (s) of the lesion (s), approximate size, extent, infiltration, distance from the esophageal-gastric transition and the pylorus, detailing the places where the biopsies were performed. 

**Table:** 

Fully agree	Partially agree	Undecided	Disagree	Strongly disagree	Total	Consensus
98%	2%	0%	0%	0%	100%	100%

####  Statement 2 

In case of high suspicion of gastric cancer and repeatedly negative biopsies collected by upper gastrointestinal endoscopy (including macrobiopsies), the diagnosis can be made through endoscopic resection or surgery. 

**Table:** 

Fully agree	Partially agree	Undecided	Disagree	Strongly disagree	Total	Consensus
58%	36%	0%	2%	4%	100%	94%

####  Statement 3 

Ultrasound upper endoscopy is not indicated when there are clear endoscopic signs that the cancer is invasive. It should be used when there is any doubt about the early aspect of GC. It allows to evaluate the degree of tumor invasion in the gastric wall and the presence of suspicious lymph nodes for metastases. 

**Table:** 

Fully agree	Partially agree	Undecided	Disagree	Strongly disagree	Total	Consensus
72%	24%	2%	2%	0%	100%	96%

####  Statement 4 

The main staging method is computed tomography of the chest, abdomen and pelvis. 

**Table:** 

Fully agree	Partially agree	Undecided	Disagree	Strongly disagree	Total	Consensus
90%	10%	0%	0%	0%	100%	100%

####  Statement 5 

Positron emission tomography *(*PET-CT) and nuclear magnetic resonance (MRI) should be used only in selected cases.

**Table:** 

Fully agree	Partially agree	Undecided	Disagree	Strongly disagree	Total	Consensus
92%	8%	0%	0%	0%	100%	100%

####  Statement 6 

PET-CT can be used in well-differentiated tumors or in the proximal third.

**Table:** 

Fully agree	Partially agree	Undecided	Disagree	Strongly disagree	Total	Consensus
44%	30%	6%	16%	4%	100%	74%

####  Statement 7 

Staging laparoscopy should be performed in cases where there is uncertainty in computed tomography regarding the presence of peritoneal carcinomatosis or when multidisciplinary treatment is planned.

**Table:** 

Fully agree	Partially agree	Undecided	Disagree	Strongly disagree	Total	Consensus
80%	18%	0%	2%	0%	100%	98%

####  Statement 8 

Peritoneal washing with oncotic cytology should be performed in all cases during staging laparoscopy and/or surgery. It may be omitted if there is frank peritoneal carcinomatosis. 

**Table:** 

Fully agree	Partially agree	Undecided	Disagree	Strongly disagree	Total	Consensus
72%	24%	4%	0%	0%	100%	96%

####  Statement 9 

Analysis of serum tumor markers (CA19.9, CEA, CA 72.4) should be performed in all cases of gastric cancer. 

**Table:** 

Fully agree	Partially agree	Undecided	Disagree	Strongly disagree	Total	Consensus
18%	38%	6%	26%	12%	100%	56%

####  Declaration 10 

Currently, the staging that must be adopted is the UICC/AJCC TNM 8^th^ edition. 

**Table:** 

Fully agree	Partially agree	Undecided	Disagree	Strongly disagree	Total	Consensus
96%	4%	0%	0%	0%	100%	100%

### Treatment statements

####  Statement 11 

Multidisciplinary therapeutic planning (surgeon, endoscopist, general clinician, oncologist, radiologist and pathologist) is recommended before starting any type of treatment. 

**Table:** 

Fully agree	Partially agree	Undecided	Disagree	Strongly disagree	Total	Consensus
70%	20%	0%	10%	0%	100%	90%

####  Statement 12 

Patients who had weight lost greater than 10% of their usual weight in the past six months should receive some form of nutritional therapy before starting any treatment.

**Table:** 

Fully agree	Partially agree	Undecided	Disagree	Strongly disagree	Total	Consensus
68%	32%	0%	0%	0%	100%	100%

####  Statement 13 

Endoscopic resection is indicated in well-differentiated adenocarcinoma tumors, restricted to the mucosa (T1a), less than 2 cm in its longest axis and not ulcerated. 

**Table:** 

Fully agree	Partially agree	Undecided	Disagree	Strongly disagree	Total	Consensus
94%	6%	0%	0%	0%	100%	100%

####  Statement 14 

Early lesions with invasion of the submucosal layer, ulcerated, diffuse type and larger than 2 cm are exception criteria for endoscopic resection and should be adopted only in patients at high surgical risk.

**Table:** 

Fully agree	Partially agree	Undecided	Disagree	Strongly disagree	Total	Consensus
76%	16%	2%	6%	0%	100%	92%

####  Statement 15 

Endoscopic submucosal dissection (ESD) is recommended as the treatment of choice for most superficial gastric tumors. 

**Table:** 

Fully agree	Partially agree	Undecided	Disagree	Strongly disagree	Total	Consensus
42%	34%	8%	8%	8%	100%	76%

####  Statement 16 

For tumors that do not meet endoscopic resection indication (T1b), surgery is indicated. In these cases, the recommended lymph node dissection is the removal of the perigastric lymph nodes (D1) in well-differentiated tumors smaller than 1.5 cm and associated with the removal of some lymph nodes in the N2 chain (D1 +) for undifferentiated tumors smaller than 1.5 cm.

**Table:** 

Fully agree	Partially agree	Undecided	Disagree	Strongly disagree	Total	Consensus
42%	34%	2%	18%	4%	100%	76%

####  Statement 17 

In stage IB-III tumors (T2-4 any N), D2 lymph node dissection is indicated. 

**Table:** 

Fully agree	Partially agree	Undecided	Disagree	Strongly disagree	Total	Consensus
90%	8%	2%	0%	0%	100%	98%

####  Statement 18 

The UICC / AJCC recommends a minimum of 15 harvested lymph nodes to allow correct staging.

**Table:** 

Fully agree	Partially agree	Undecided	Disagree	Strongly disagree	Total	Consensus
78%	14%	0%	6%	2%	100%	92%

####  Statement 19 

D2 lymphadenectomy recommends at least 25 harvested lymph nodes. 

**Table:** 

Fully agree	Partially agree	Undecided	Disagree	Strongly disagree	Total	Consensus
54%	22%	4%	18%	2%	100%	76%

####  Statament 20 

It is recommended at the end of each operation that a member of the surgical team send the surgical specimen for the pathological analysis with all the separate and identified lymph node chains.

**Table:** 

Fully agree	Partially agree	Undecided	Disagree	Strongly disagree	Total	Consensus
72%	18%	2%	8%	0%	100%	90%

####  Statement 21 

Lymphadenectomies more extended than D2 (D2 + or D3) should be reserved only in selected cases.

**Table:** 

Fully agree	Partially agree	Undecided	Disagree	Strongly disagree	Total	Consensus
84%	8%	2%	6%	0%	100%	92%

####  Statement 22 

Subtotal gastrectomy should be performed on distal tumors or in cases where the proximal margin is at least 5 cm between the tumor and the esophagogastric transition. 

**Table:** 

Fully agree	Partially agree	Undecided	Disagree	Strongly disagree	Total	Consensus
70%	26%	2%	2%	0%	100%	96%

####  tatement 23 

In diffuse tumors, a proximal margin of at least 8 cm is recommended.

**Table:** 

Fully agree	Partially agree	Undecided	Disagree	Strongly disagree	Total	Consensus
34%	38%	8%	16%	4%	100%	72%

####  Statement 24 

In tumors invading the distal esophagus, the resection margin must be confirmed by frozen biopsy.

**Table:** 

Fully agree	Partially agree	Undecided	Disagree	Strongly disagree	Total	Consensus
72%	22%	2%	2%	2%	100%	94%

####  Statement 25 

Total gastrectomy is recommended for proximal tumors and early multicentric tumors. 

**Table:** 

Fully agree	Partially agree	Undecided	Disagree	Strongly disagree	Total	Consensus
74%	26%	0%	0%	0%	100%	100%

####  Statement 26 

Splenectomy should be performed only in advanced tumors from the greater curvature of the proximal stomach, when there is invasion of the spleen or if there is evident lymph node involvement of the splenic hilum. 

**Table:** 

Fully agree	Partially agree	Undecided	Disagree	Strongly disagree	Total	Consensus
76%	20%	4%	0%	0%	100%	96%

####  Statement 27 

Patients with unresectable or marginally resectable lesions may be candidates for conversion therapy, which consists of chemotherapy followed by surgery to achieve R0 resection*.*

**Table:** 

Fully agree	Partially agree	Undecided	Disagree	Strongly disagree	Total	Consensus
78%	20%	0%	2%	0%	100%	98%

####  Statement 28 

Duodenopancreatectomy can be indicated in cases of locally advanced gastric cancer, T4N0-2M0, young patients and with low surgical risk.

**Table:** 

Fully agree	Partially agree	Undecided	Disagree	Strongly disagree	Total	Consensus
58%	32%	4%	6%	0%	100%	90%

####  Statement 29 

Hepatectomy is indicated in infiltrative liver tumors (T4b) without peritoneal carcinomatosis. 

**Table:** 

Fully agree	Partially agree	Undecided	Disagree	Strongly disagree	Total	Consensus
68%	30%	0%	2%	0%	100%	98%

####  Statement 30 

Patients with single liver metastasis may be eligible for surgery after a multidisciplinary evaluation.

**Table:** 

Fully agree	Partially agree	Undecided	Disagree	Strongly disagree	Total	Consensus
62%	24%	8%	6%	0%	100%	86%

####  Statement 31 

The combined resection of adjacent or multivisceral organs can be performed, as long as the patient is in good clinical condition and, preferably, R0 resection is achieved.

**Table:** 

Fully agree	Partially agree	Undecided	Disagree	Strongly disagree	Total	Consensus
88%	8%	0%	4%	0%	100%	96%

####  Statement 32 

In patients considered M1, palliative gastric resection may occasionally be performed in cases of obstruction, bleeding or perforation.

**Table:** 

Fully agree	Partially agree	Undecided	Disagree	Strongly disagree	Total	Consensus
90%	10%	0%	0%	0%	100%	100%

####  Statement 33 

Palliative gastric resection in asymptomatic patients is not indicated as the first treatment approach*.*

**Table:** 

Fully agree	Partially agree	Undecided	Disagree	Strongly disagree	Total	Consensus
70%	16%	4%	10%	0%	100%	86%

####  Statement 34 

Partial omentectomy (up to 3-5 cm from the gastroepiploic arcade) can be performed on T1/T2 tumors and total omentectomy must be performed on T3/T4 tumors.

**Table:** 

Fully agree	Partially agree	Undecided	Disagree	Strongly disagree	Total	Consensus
48%	30%	14%	6%	2%	100%	78%

####  Statement 35 

Bursectomy should be performed only on T4 tumors arising from the posterior gastric wall. 

**Table:** 

Fully agree	Partially agree	Undecided	Disagree	Strongly disagree	Total	Consensus
48%	32%	8%	12%	0%	100%	80%

####  Statement 36 

Prophylactic total gastrectomy is indicated in confirmed familial hereditary gastric cancer with the CDh1 gene mutation. 

**Table:** 

Fully agree	Partially agree	Undecided	Disagree	Strongly disagree	Total	Consensus
72%	28%	0%	0%	0%	100%	100%

####  Statement 37 

Laparoscopic subtotal gastrectomy can be performed in distal third early GC. 

**Table:** 

Fully agree	Partially agree	Undecided	Disagree	Strongly disagree	Total	Consensus
90%	8%	2%	0%	0%	100%	98%

####  Statement 38 

Laparoscopic subtotal gastrectomy can be performed in distal third advanced gastric cancer. 

**Table:** 

Fully agree	Partially agree	Undecided	Disagree	Strongly disagree	Total	Consensus
52%	40%	8%	0%	0%	100%	92%

####  Statement 39 

Laparoscopic total gastrectomy can be performed in upper third early gastric cancer. 

**Table:** 

Fully agree	Partially agree	Undecided	Disagree	Strongly disagree	Total	Consensus
62%	28%	8%	2%	0%	100%	90%

####  Statement 40 

Laparoscopic total gastrectomy can be performed in upper third advanced GC.

**Table:** 

Fully agree	Partially agree	Undecided	Disagree	Strongly disagree	Total	Consensus
38%	38%	16%	8%	0%	100%	76%

####  Statement 41 

The use of the robotic platform has the same indications and results as laparoscopy.

**Table:** 

Fully agree	Partially agree	Undecided	Disagree	Strongly disagree	Total	Consensus
64%	26%	8%	2%	0%	100%	90%

####  Statement 42 

In Siewert type III adenocarcinomas, the standard surgery is total gastrectomy with distal esophagectomy. 

**Table:** 

Fully agree	Partially agree	Undecided	Disagree	Strongly disagree	Total	Consensus
82%	14%	2%	2%	0%	100%	96%

####  Statement 43 

In Siewert type II adenocarcinomas, the standard surgery is transthoracic esophagectomy (thoracoscopy) with proximal gastrectomy and gastric conduit. 

**Table:** 

Fully agree	Partially agree	Undecided	Disagree	Strongly disagree	Total	Consensus
32%	32%	10%	22%	4%	100%	64%

####  Statement 44 

In Siewert type I adenocarcinomas, the standard surgery is transthoracic esophagectomy (thoracoscopy) with proximal gastrectomy and gastric conduit.

**Table:** 

Fully agree	Partially agree	Undecided	Disagree	Strongly disagree	Total	Consensus
74%	18%	4%	4%	0%	100%	92%

####  Statement 45 

Transmediastinal esophagectomy should be reserved for patients with poor or borderline clinical conditions and/or inability to access the thoracic cavity.

**Table:** 

Fully agree	Partially agree	Undecided	Disagree	Strongly disagree	Total	Consensus
36%	42%	6%	14%	2%	100%	78%

####  Statement 46 

It is recommended that following an esophagectomy, the esophagogastrostomy should be performed preferably in the cervical region. 

**Table:** 

Fully agree	Partially agree	Undecided	Disagree	Strongly disagree	Total	Consensus
66%	24%	6%	2%	2%	100%	90%

####  Statement 47 

Routine abdominal drain(s) are recommended for all gastric resections. 

**Table:** 

Fully agree	Partially agree	Undecided	Disagree	Strongly disagree	Total	Consensus
36%	34%	6%	22%	2%	100%	70%

####  Statement 48 

The duodenal stump should preferably be closed using mechanical suture.

**Table:** 

Fully agree	Partially agree	Undecided	Disagree	Strongly disagree	Total	Consensus
58%	26%	6%	8%	2%	100%	84%

####  Statement 49 

There is no clear scientific evidence that reinforcement of the duodenal stump stapling line reduces the incidence of fistulas. 

**Table:** 

Fully agree	Partially agree	Undecided	Disagree	Strongly disagree	Total	Consensus
60%	24%	12%	4%	0%	100%	84%

####  Statement 50 

In subtotal and total gastrectomies, digestive transit should preferably be reconstructed by Roux-en-Y derivation.

**Table:** 

Fully agree	Partially agree	Undecided	Disagree	Strongly disagree	Total	Consensus
84%	12%	4%	0%	0%	100%	96%

####  Statement 51 

Gastrojejunostomy and esophagojejunostomy should preferably be performed with mechanical suture. 

**Table:** 

Fully agree	Partially agree	Undecided	Disagree	Strongly disagree	Total	Consensus
30%	40%	10%	16%	4%	100%	70%

####  Statement 52 

After gastric resection, oral feeding should be started as soon as the patient has conditions and the intestinal transit is restored.

**Table:** 

Fully agree	Partially agree	Undecided	Disagree	Strongly disagree	Total	Consensus
48%	35%	2%	6%	9%	100%	83%

### Chemoradiotherapy statements

####  Statement 53 

Perioperative chemotherapy (before and after surgery) is indicated for stage ≥IB resectable tumors of the distal third. 

**Table:** 

Fully agree	Partially agree	Undecided	Disagree	Strongly disagree	Total	Consensus
34%	34%	6%	20%	6%	100%	68%

####  Statement 54 

Perioperative chemotherapy (before and after surgery) is indicated for stage ≥I resectable tumors of the middle and proximal third.

**Table:** 

Fully agree	Partially agree	Undecided	Disagree	Strongly disagree	Total	Consensus
38%	40%	4%	18%	0%	100%	78%

####  Statement 55 

Stage ≥IB patients who underwent surgery without perioperative chemotherapy (up front surgery) have an indication for adjuvant chemotherapy.

**Table:** 

Fully agree	Partially agree	Undecided	Disagree	Strongly disagree	Total	Consensus
44%	36%	6%	10%	4%	100%	80%

####  Statement 56 

Adjuvant radiotherapy is recommended in cases with an indication for adjuvant chemotherapy and who did not have an adequate lymph node dissection during surgery.

**Table:** 

Fully agree	Partially agree	Undecided	Disagree	Strongly disagree	Total	Consensus
38%	44%	8%	10%	0%	100%	82%

####  Statement 57 

Patients with metastatic gastric cancer, in good clinical condition, have an indication for palliative chemotherapy.

**Table:** 

Fully agree	Partially agree	Undecided	Disagree	Strongly disagree	Total	Consensus
76%	18%	6%	0%	0%	100%	94%

####  Statement 58 

Patients with metastatic gastric cancer who respond well to palliative chemotherapy and have little residual disease are candidates for conversion therapy with the aim of achieving an R0 resection.

**Table:** 

Fully agree	Partially agree	Undecided	Disagree	Strongly disagree	Total	Consensus
48%	36%	6%	8%	2%	100%	84%

####  Statement 59 

Hyperthermic intraperitoneal chemotherapy (HIPEC) should be used only in research protocols, as there is still no consistent evidence of its real benefit.

**Table:** 

Fully agree	Partially agree	Undecided	Disagree	Strongly disagree	Total	Consensus
74%	12%	6%	6%	2%	100%	86%

####  Statement 60 

Metastatic gastric cancer patients HER-2 positive are indicated for target therapy treatment (monoclonal antibody) associated with palliative chemotherapy. 

**Table:** 

Fully agree	Partially agree	Undecided	Disagree	Strongly disagree	Total	Consensus
72%	20%	8%	0%	0%	100%	92%

####  Statement 61 

Immunotherapy for patients with metastatic gastric cancer should be used only in research protocols, as there is still no consistent evidence of its real benefit. 

**Table:** 

Fully agree	Partially agree	Undecided	Disagree	Strongly disagree	Total	Consensus
42%	28%	8%	16%	6%	100%	70%

### Follow-up statements

####  Statement 62 

Patients with metastatic gastric cancer who have not responded to palliative chemotherapy or in poor clinical conditions, should receive only palliative care with best support of care.

**Table:** 

Fully agree	Partially agree	Undecided	Disagree	Strongly disagree	Total	Consensus
84%	12%	2%	2%	0%	100%	96%

####  Statement 63 

Patients undergoing radical surgery or after adjuvant therapy should not be followed due to the high cost and because there is no evidence that the follow-up improves survival. 

**Table:** 

Fully agree	Partially agree	Undecided	Disagree	Strongly disagree	Total	Consensus
4%	14%	2%	36%	44%	100%	18%

####  Statement 64 

Patients submitted to radical surgery can be followed through abdominal ultrasound, due to its accessibility and low cost.

**Table:** 

Fully agree	Partially agree	Undecided	Disagree	Strongly disagree	Total	Consensus
10%	28%	6%	42%	14%	100%	38%

####  Statement 65 

In the postoperative period of patients submitted to radical surgery, the upper endoscopy is indicated when there is clinical suspicion of recurrence and digestive symptoms.

**Table:** 

Fully agree	Partially agree	Undecided	Disagree	Strongly disagree	Total	Consensus
48%	30%	0%	20%	2%	100%	78%

####  Statement 66 

The long-term follow-up should be offered to patients undergoing radical surgery or after the end of adjuvant therapy for nutritional and psychological control and support, early detection of recurrence, treatment of complications and data collection. 

**Table:** 

Fully agree	Partially agree	Undecided	Disagree	Strongly disagree	Total	Consensus
88%	12%	0%	0%	0%	100%	100%

####  Statement 67 

The attempt of surgical resection in patients with single local recurrence and low surgical risk can be considered in selected cases. 

**Table:** 

Fully agree	Partially agree	Undecided	Disagree	Strongly disagree	Total	Consensus
78%	20%	0%	0%	2%	100%	98%

## DISCUSSION

The word consensus originates from the Latin (consensus) and, by definition, means the agreement or uniformity of opinions, thoughts, feelings and beliefs of the majority or all of the members of a collective. Consensus are quite common in medicine. They are usually performed by experts in certain areas and consist of creating diagnostic and treatments guidelines for certain diseases^7^.

Several international societies have published their respective consensus and guidelines^2^ ^,^ ^3^ ^,^ ^4^ ^,^ ^8^ ^,^ ^12^ ^,^ ^13^ ^,^ ^19^ ^,^ ^20^ ^,^ ^22^. In fact, it is paramount that each country has its own consensus. Although GC has similar characteristics between different races, there are peculiarities between different countries that must be addressed according to local conditions. These include the incidence of the disease, sanitary conditions, eating habits, cultural aspects, accessibility to diagnostic methods and treatment, among others. These differences can be seen in our country. Due to its enormous size, there is a huge difference between the aspects mentioned between the regions of Brazil. Therefore, it is essential that each location seeks to adapt the information contained in this consensus with its own reality, always seeking early diagnosis and the most effective treatment possible.

Unlike the I Consensus published in 2013^21^, in which the participants had the option to answer only yes or no to the questions, the authors of this consensus chose to provide more options for answers. This is because GC is a complex disease, with multiple factors that may influence its management. In short, often more than one path considered to be correct can be followed. In addition, this will allow, in future publications, each statement to be commented on based on evidence from the latest medical literature worldwide.

## CONCLUSION

The treatment of gastric cancer has evolved considerably in recent years. This consensus gathers the answers of the questionnaire prepared by ABCG of several specialists in gastric cancer treatment in Brazil. This is the commitment of the Brazilian Gastric Cancer Association: to disseminate knowledge and allow professionals to achieve better outcomes in terms of patients’ survival and quality of life.
